# Oxytetracycline loading onto Cu–CuO/Cu_2_O nanocomposites (NCs): structural characterization and enhanced antimicrobial activity

**DOI:** 10.1039/d6ra06019f

**Published:** 2026-10-16

**Authors:** Asmaa H. Hammadi, Saba Abdulmunem Habeeb, Lena Fadhil Hamza, Amer Al-Nafiey, Rafal Al-Assaly

**Affiliations:** a Department of Pharmaceutics, College of Pharmacy, University of Babylon Babylon Al Hillah Iraq; b Department of Pharmaceutical Chemistry, College of Pharmacy, University of Babylon Babylon Al Hillah Iraq; c Department of Pharmacology and Toxicology, College of Pharmacy, University of Babylon Babylon Al Hillah Iraq; d Department of Laser Physics, College of Science for Women, University of Babylon Babylon Al Hillah Iraq amer76z@yahoo.com; e Department of Clinical Laboratory Sciences, College of Pharmacy, University of Babylon Babylon Al Hillah Iraq

## Abstract

In this study, the chemical reduction technique was utilized to create a three-phase nanocomposite system including metallic copper (Cu^0^), cupric oxide (CuO, Cu^2+^), and cuprous oxide (Cu_2_O, Cu^+^) inside a single matrix in one step. The Cu–CuO/Cu_2_O nanocomposite NCs were analyzed by XRD, SEM, EDX, and FT-IR to study the structural and chemical properties. The XRD analysis elucidated the crystallinity of the Cu–CuO/Cu_2_O NPs, revealing multiple patterns of copper, including Cu, CuO, and Cu_2_O, with a mean particle size of 21.2 nm. SEM + EDX show the morphology of the Cu–CuO/Cu_2_O NCs with an average grain diameter ≈30 nm and a distribution range of 5–53 nm. The SEM images proved loading of the Cu–CuO/Cu_2_O NCs with the Oxytetracycline (OTC) drug. Oxytetracycline (OTC) was delivered with the Cu–CuO/Cu_2_O NCs for developing resistance against microbial pathogens. The results showed that the minimum inhibitory concentration of the OTC@Cu–CuO/Cu_2_O NCs against the Gram-negative bacteria *E. coli* was 62 µg mL^−1^ with an IC_50_ of 29.4 µg mL^−1^. While the minimum inhibitory concentration of OTC@Cu–CuO/Cu_2_O NPs against the Gram-positive bacteria *Staph. aureus* was 62 µg mL^−1^ with an IC_50_ of 21.8 µg mL^−1^. Additionally, the minimum inhibitory concentration of OTC@Cu–CuO/Cu_2_O NCs *versus Candida albicans* was 250 µg mL^−1^, with an IC_50_ of 37.5 µg mL^−1^. It is therefore found that this study could be used in the formulation of nano@drug conjugates that serve as antimicrobial agents in various fields of medical and pharmaceutical research.

## Abstract 2

In this study, the chemical reduction technique was utilized to create a three-phase nanocomposite system including metallic copper (Cu^0^), cupric oxide (CuO, Cu^2+^), and cuprous oxide (Cu_2_O, Cu^+^) inside a single matrix in one step.

## Graphical abstract

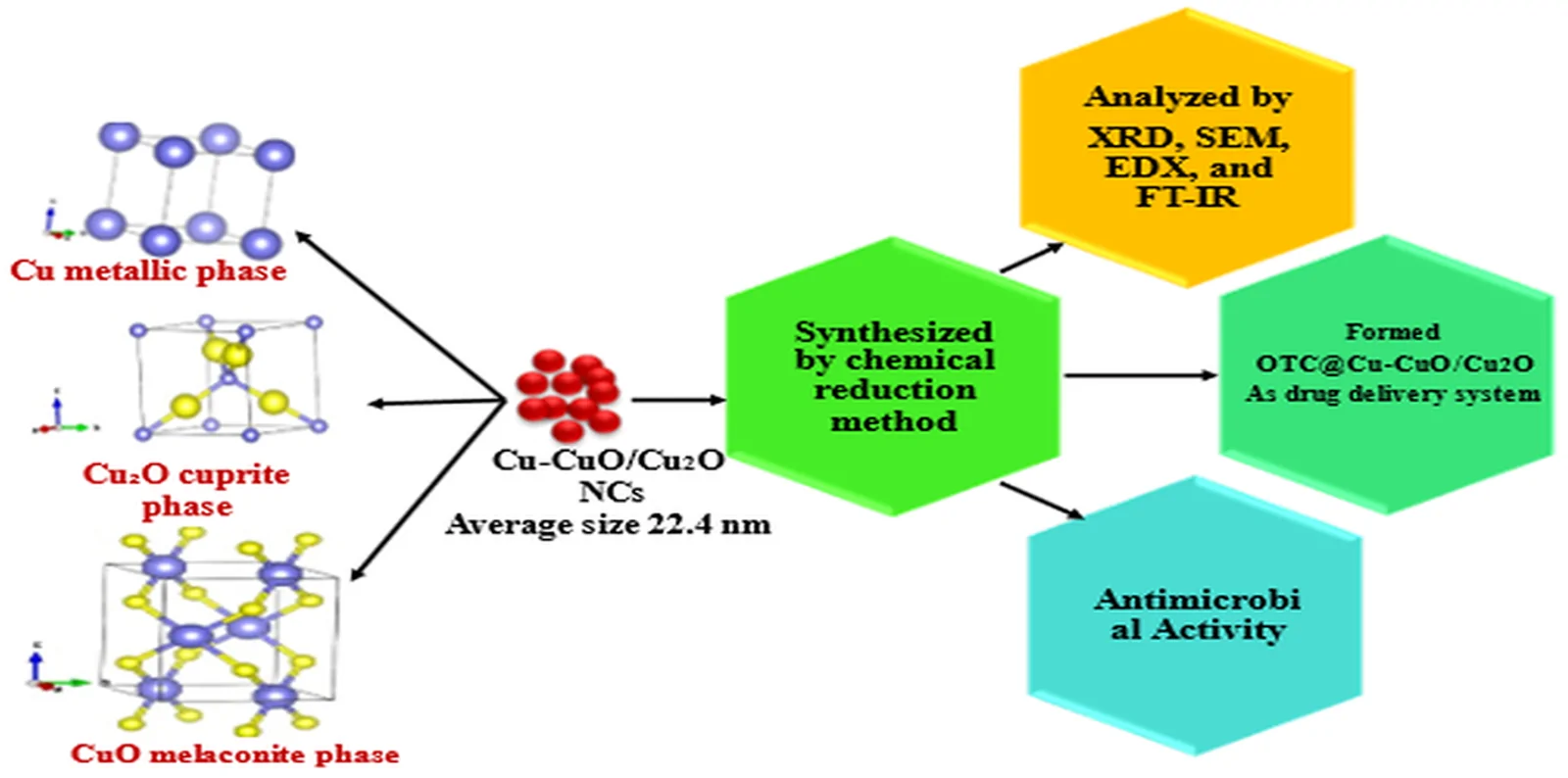

## 1 Introduction

Antimicrobial resistance has become a major global challenge that reduces the effectiveness of standard antibiotic therapies and increases morbidity, mortality, and healthcare burden. The extensive misuse and overuse of antibiotics in clinical and agricultural settings have accelerated the spread of resistant Gram-positive and Gram-negative bacteria, as well as pathogenic fungi. Consequently, many conventional antimicrobial agents have shown declining efficacy against multidrug-resistant strains.^1,2^

Most antibiotic treatments still rely on monotherapy mechanisms that target specific microbial pathways. Although initially effective, microorganisms rapidly develop resistance through mutation, enzymatic degradation, efflux pump activation, or modification of antibiotic binding sites. These adaptations frequently necessitate elevated therapeutic doses, potentially heightening toxicity and adverse effects without ensuring favorable treatment results.^2,3^ The limited pipeline of novel antibiotics further underscores the urgency of identifying alternative strategies to preserve or restore the efficacy of existing drugs.^1^

Nanoparticle-based antimicrobial systems have emerged as promising tools to address these limitations. Due to their nanoscale dimensions, nanoparticles exhibit high surface reactivity and strong interactions with microbial membranes. They function both as drug carriers and as direct antimicrobial agents through membrane disruption, metal ion release, and reactive oxygen species (ROS) generation. This multi-mechanistic mode of action reduces the likelihood of resistance development, in contrast to conventional antibiotics that typically act on a single intracellular target.^2^

Nanocarrier-assisted delivery can enhance antibiotic performance by improving drug solubility, stability, and accumulation at infection sites. This approach may reduce the required antibiotic dose and limit selective pressure that drives resistance. Additionally, metal and metal oxide nanoparticles help kill bacteria in different ways, working together with antimicrobial drugs for better results (Scheme 1).^3^

**Scheme 1:**
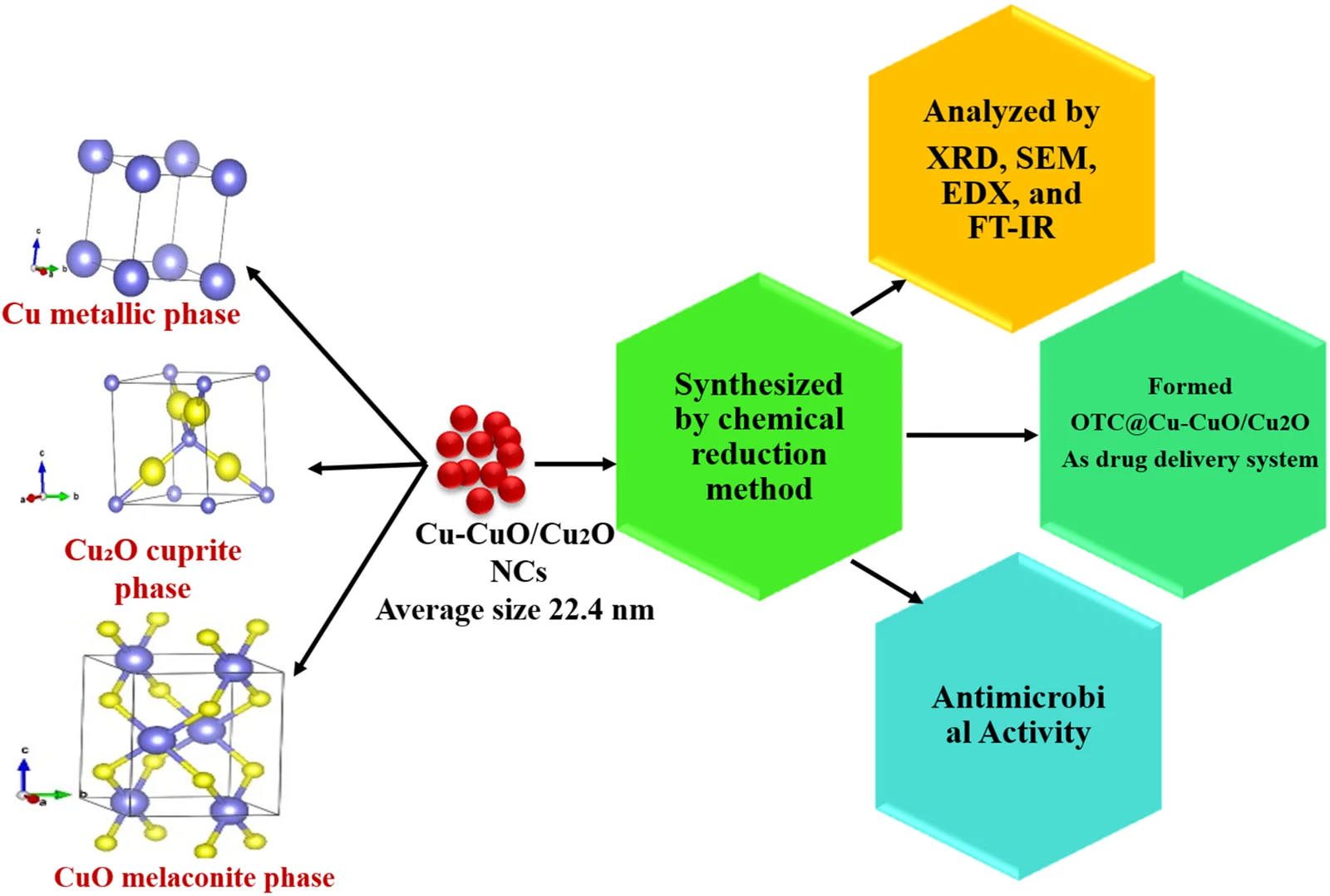
Illustrates the phases, analysis, and applications of the Cu–CuO/Cu_2_O NCs that were synthesized through chemical reduction.

Copper and copper oxide nanoparticles have attracted considerable attention owing to their broad-spectrum antimicrobial activity, low cost, and ready availability. Cu, CuO, and Cu_2_O nanostructures inhibit bacterial and fungal growth through both oxidative and non-oxidative mechanisms. Mixed-phase Cu–CuO/Cu_2_O nanocomposites are of particular interest, as the coexistence of multiple copper phases may enhance antimicrobial efficacy through enhanced ion release and redox interactions.^4^

Table 1 summarizes various synthesis routes for Cu–CuO/Cu_2_O nanocomposites and their reported applications across different fields. This comparative overview highlights the versatility of copper-based hybrid systems and motivates their investigation as carriers and enhancers for antibiotic delivery.

**Table 1:** List of key applications of Cu–CuO/Cu_2_O synthesized *via* different methods

Nanocomposite	Synthesis method	Structure analysis	Application	Ref.
Cu–CuO/Cu_2_O	Chemical reduction	XRD, SEM, EDX, FT-IR	Drug delivery & microbial activity	Our work
Cu–CuO/Cu_2_O	Green chemistry	UV-Vis, PL, SEM, HRTEM, Raman, FT-IR, TGA	Study electrochemical properties	5
Cu–CuO/Cu_2_O	Physical method (laser)	UV-Vis, EDX, DRS	Anti-microbial activity	6
Cu_2_O–CuO–Cu–C	Coupling with aryl azides	XRD, FT-IR, HR-TEM, XPS, EDX	—	7
Cu–CuO/Cu_2_O	Hydrothermal method	XRD, SEM, HR-TEM, SEM, SAED, XPS	Photocatalytic	8
(Cu/CuO/Cu_2_O) microspheres	Hydrothermal method	SEM, XRD, FT-IR	Dyes removal	9
Cu–CuO and Cu–Cu_2_O	Electro-explosion of wire method	XRD, FESEM, FT-IR	Study physical properties	10

On the other hand, oxytetracycline (OTC) is a broad-spectrum tetracycline antibiotic widely used against Gram-positive and Gram-negative infections, as seen in the chemical structure of OTC in Fig. 1. However, its clinical effectiveness has declined due to resistance mechanisms such as efflux transport and ribosomal protection. Recent studies have therefore explored nanoparticle-based OTC delivery platforms, including mesostructured silica and pH-responsive carriers, to improve antibacterial outcomes.^10,11^

**Fig. 1:**
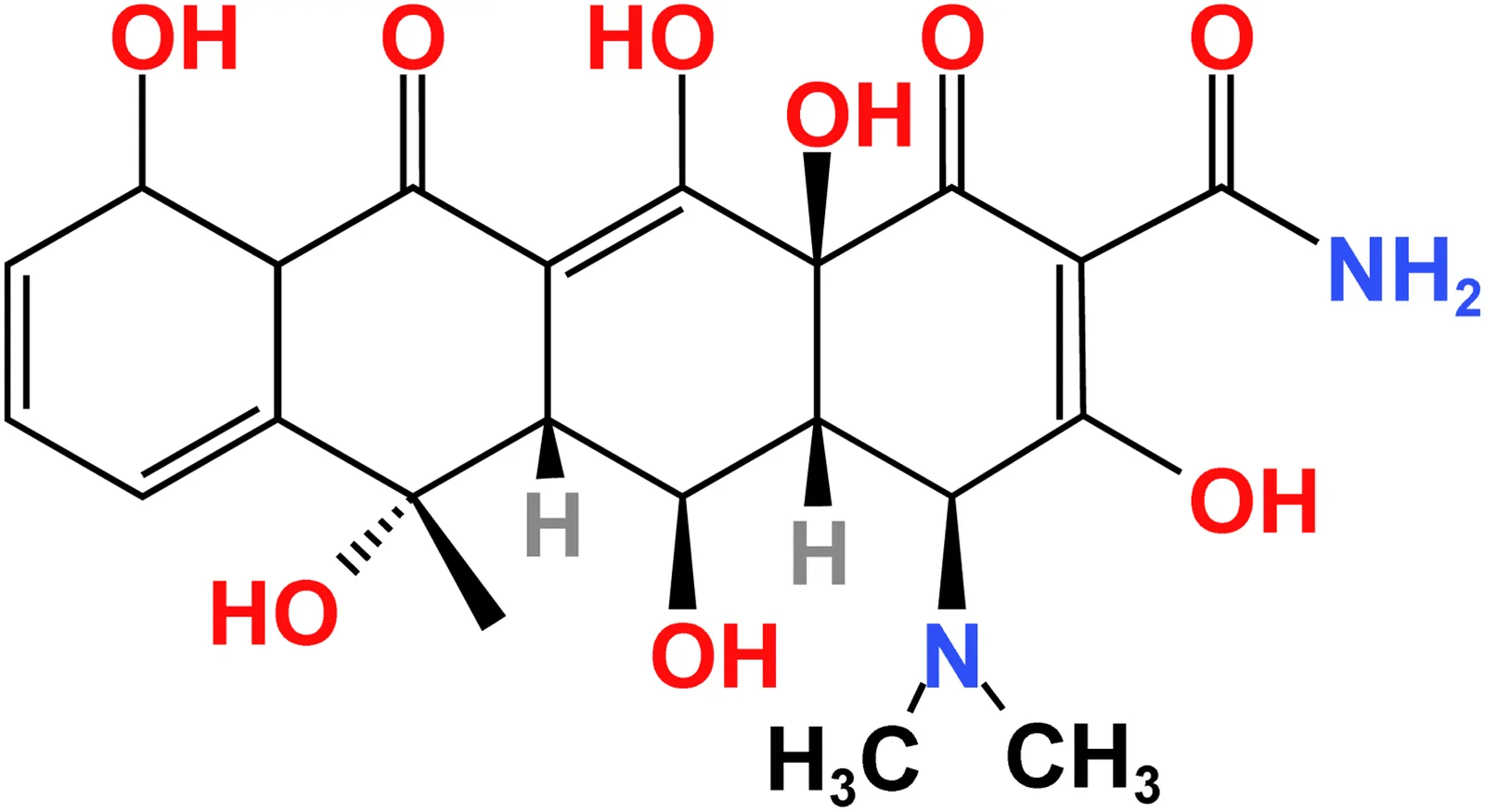
Chemical structure of oxytetracycline (OTC). The copyright of the chemical structure of oxytetracycline (OTC) in the Journal of the *American Chemical Society.*^12^

The aim of this study had been used the chemical reduction method to generate a three-phase nanocomposites system of metallic copper (Cu^0^), cupric oxide (CuO, Cu^2+^), and cuprous oxide (Cu_2_O, Cu^+^) in one product (Cu–CuO/Cu_2_O NCs). Using this copper-infused nanocomposites as a nanocarrier for Oxytetracycline (OTC) is a new way to protect and increase bioavailability. Cu–CuO/Cu_2_O nanocomposites were synthesized *via* chemical reduction and loaded with OTC to form OTC@Cu–CuO/Cu_2_O hybrid systems. Structural and morphological characterization was performed using X-ray diffraction (XRD), scanning electron microscopy coupled with energy-dispersive spectroscopy (SEM/EDX), and Fourier-transform infrared spectroscopy (FTIR). The effectiveness of free OTC, Cu–CuO/Cu_2_O nanocomposites, and OTC@Cu–CuO/Cu_2_O was tested against different types of bacteria and fungus. The study aims to show how mixed-phase copper nanocomposites make OTC more effective and to highlight their potential for delivering antimicrobial treatments.

## 2 Experimental part

### 2.1 Chemicals

Copper (Cu^2+^) pentahydrate sulfate (CuSO_4_·5(H_2_O)), polymer polyvinylpyrrolidone PVP (C_6_H_9_NO)_*n*_, ascorbic acid (C_6_H_8_O_6_), and sodium hydroxide (NaOH) were purchased from Merck, Germany. Deionized water was used for all the experiments. Muller–Hinton agar, Muller–Hinton broth, McFarland 0.67, and a Biotek 800ST plate reader (Biotek, USA) for antimicrobial activity measurements.

### 2.2 Synthesis of the Cu–CuO/Cu_2_O NCs

As seen in Fig. 2, the Cu–CuO/Cu_2_O NCs synthesized by the chemical reduction method are as follows: first, at room temperature CuSO_4_·5(H_2_O) is used as the precursor salt and PVP (C_6_H_9_NO)n as the capping agent. To prepare, add (0.2 M) of CuSO_4_·5(H_2_O) solution to (120 mL) of (1.2%) PVP (C_6_H_9_NO)_*n*_. Stir vigorously for 0.5 h. Subsequently, drop 50 mL of a 0.4 M solution of ascorbic acid (vitamin C) with a controlled pH ≈ 5 as a reducing reagent into the synthesis mixture while quickly mixing. Subsequently, gradually introduce 60 mL of 1 molar NaOH solution in pH ≈ 9 as a precipitating agent to the resultant solution while maintaining continuous stirring and warming at 80 °C for a duration of (2 h). The reaction mixture underwent a color transition from yellow to reddish-orange. The precipitate was cooled, centrifuged, filtered, washed with (70%) ethanol, dried at (90 °C) for 120 min, and then left at room temperature overnight. Until the analysis stage, the nano-production samples were kept in a glass container, protected from sunlight and stored in a dry location.^11^

**Fig. 2:**
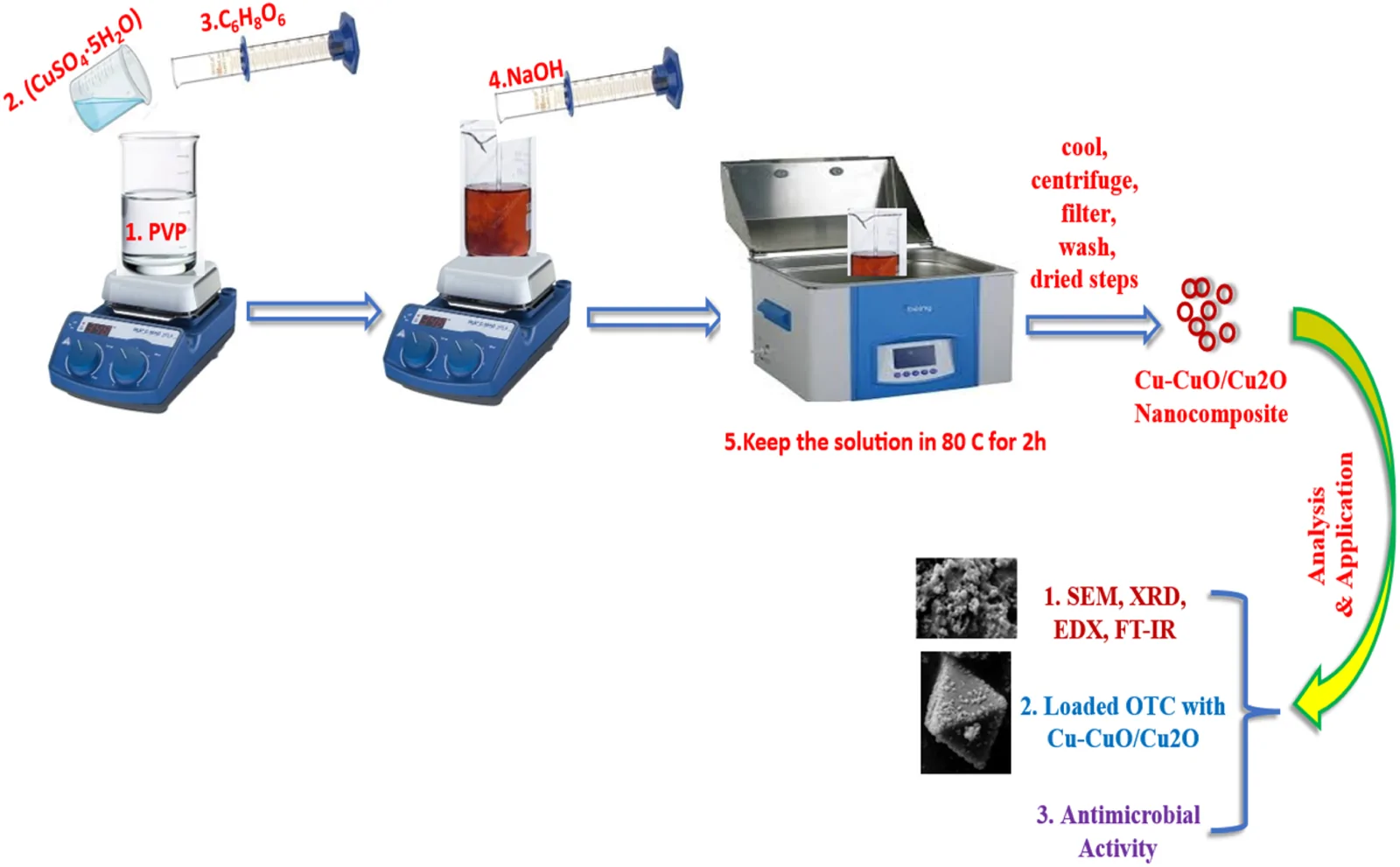
Chemical reduction steps for synthesis of Cu–CuO/Cu_2_O NCs.

During the chemical reduction method, the Cu–CuO/Cu_2_O NCs had passed by mechanistic roles, illustrated in 4 steps.^11,14^

I. Desolation in water step:
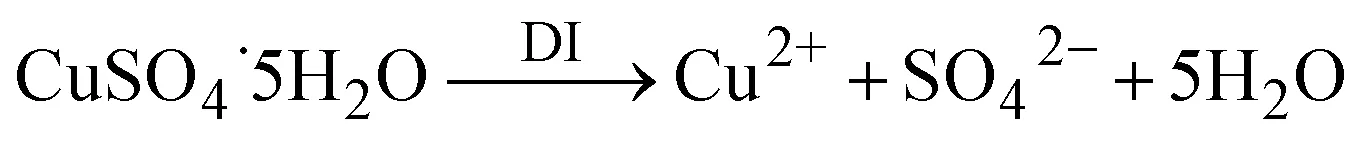


II. Complexation/stabilization by polyvinylpyrrolidone (PVP) step:
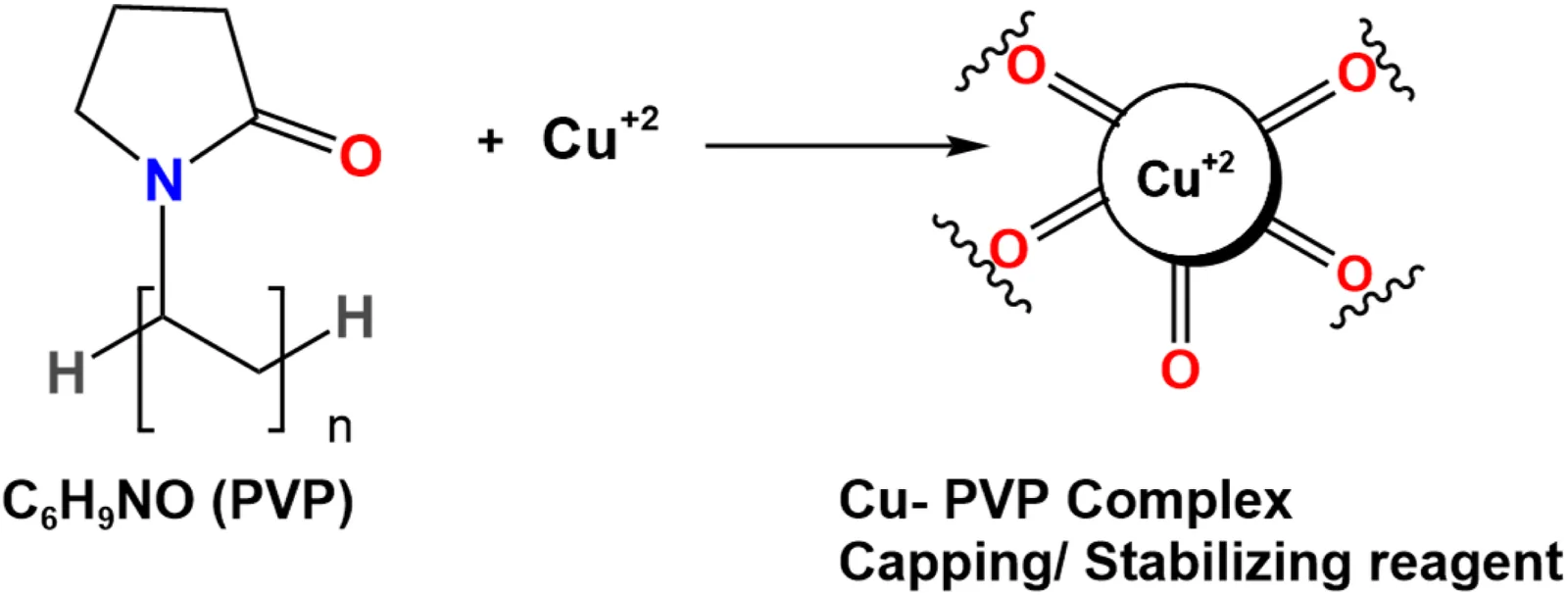


III. Reduction by ascorbic acid (vitamin C):
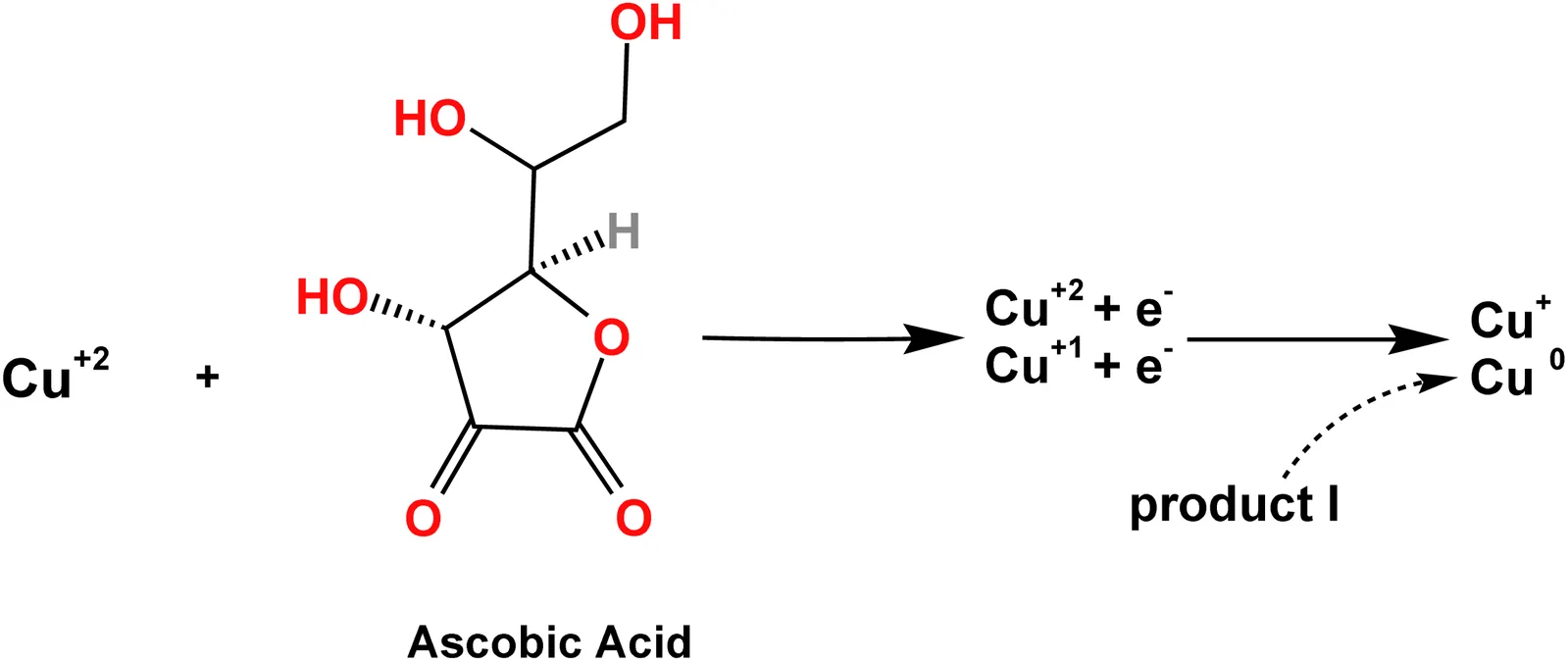


IV. Formation copper oxides by NaOH (pH ≈ 9) step:
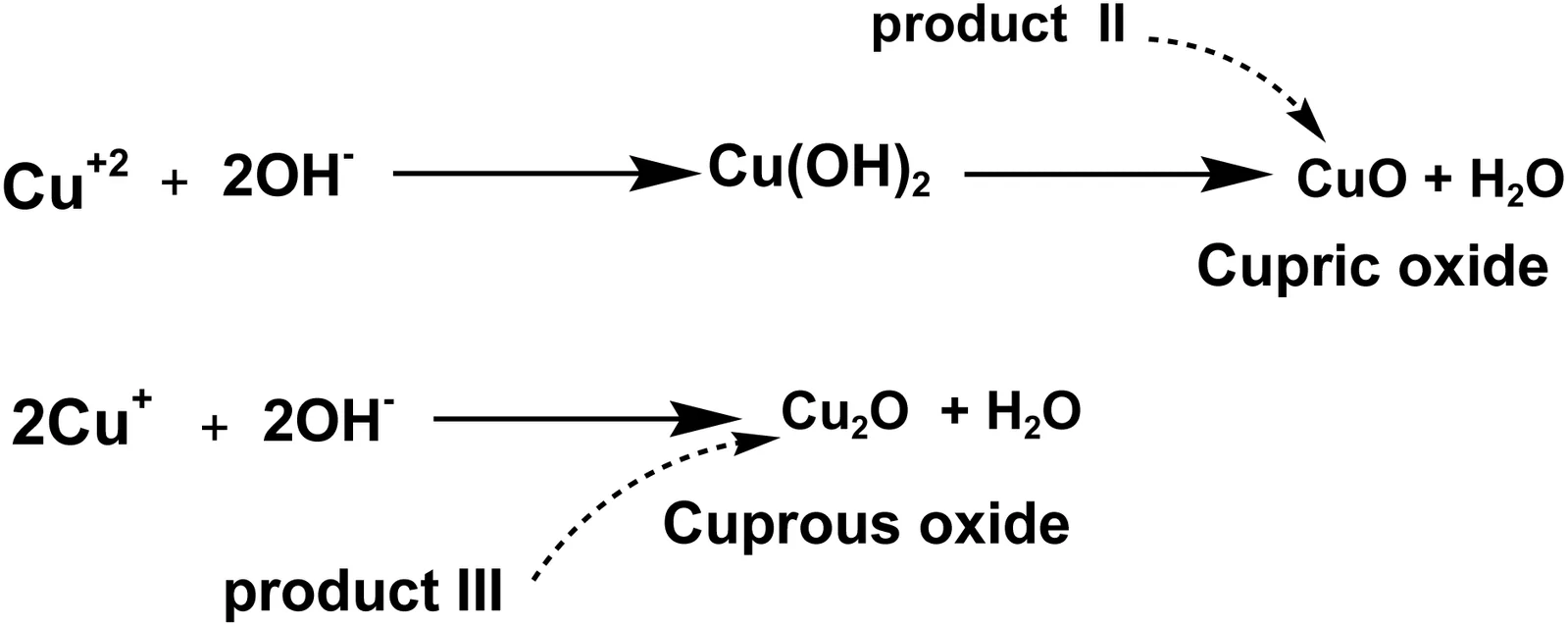


V. Nucleation and growth of Cu–Cu_2_O–CuO step:
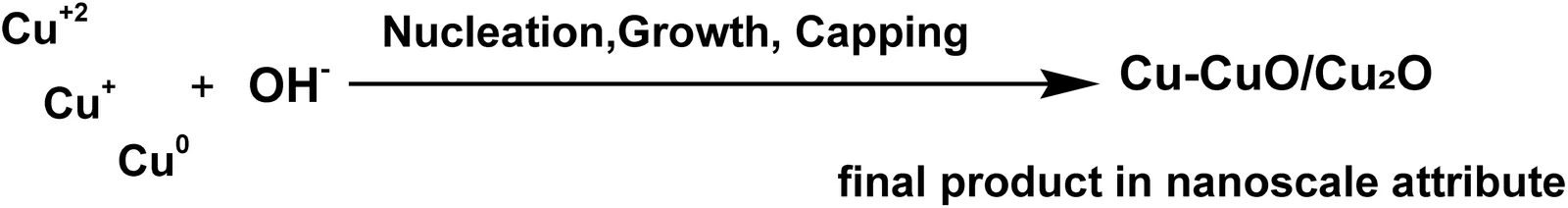


### 2.3 Delivery oxytetracycline (OTC) on Cu–CuO/Cu_2_O NCs

To prepare the OTC@Cu–CuO/Cu_2_O, (24.5 mg) of Cu–CuO/Cu_2_O NCs was added to (50 mL) of distilled water, and then (30 mg) of OTC was added to the mixture. It served as the OTC carrier. The reaction was subjected to sonication for a period of 30 min. Following that, it was processed with continuous stirring for a period of (24 h). All steps of the experiment were executed at room temperature in a dark medium with no constraints.^13^

### 2.4 Structural analysis of Cu–CuO/Cu_2_O NCs

The powder samples were encapsulated within KBr pellets for analytical purposes. The technical data derived from the Fourier transform infrared (FT-IR) analysis were acquired using an FTIR spectrometer (MAGNA-560). The X-ray diffraction patterns of Cu–CuO/Cu_2_O NCs were acquired at ambient temperature utilizing a Rigaku D-max C III X-ray diffractometer, employing Ni-filtered Kα radiation over the 2*θ* range of 5–80°. The diameter of the nanoparticles was ascertained by employing the Debye–Scherrer eqn (1).1
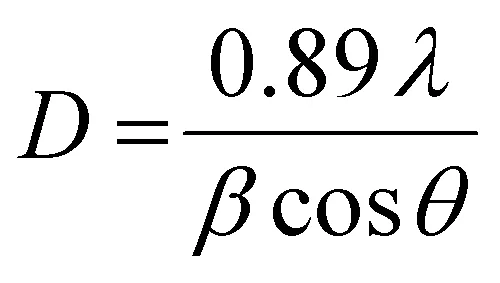


The wavelength of X-rays is symbolized by *λ*, whereas the full width at half maximum intensity is symbolized by *β*.^14^ The structural characteristics and morphological attributes of the Cu–CuO/Cu_2_O NCs, both before and after the loading with OTC, were analyzed utilizing scanning electron microscopy (SEM + EDX) techniques, with the Philips PW1730 microscope functioning at an accelerating voltage of 10 kV. The assessment of particle size distribution was conducted through the processing of SEM images utilizing NIH Image J analysis software. The mean particle size was determined through the measurement of 65 randomly selected particles.

### 2.5 Microbial activity

Microbial suspensions of *Escherichia coli* (ATCC 8739), *Staphylococcus aureus* (ATCC 29213), and isolates of *Candida albicans* were obtained from ten different 24-hour-old colonies grown on Muller–Hinton agar (MHA). The colonies were transferred into 10 ml tubes containing (2 ml) of sterile water, and the absorbance at 600 nm (OD 600) was measured utilizing a Biotek 800ST plate reader. The bacterial suspension was actually standardized to a 1.0 McFarland standard (OD_600_ ≈ 0.236), which we calibrate in our lab using CFU counts to ensure a density of about 3×10^8^ CFU per mL. This stock was then diluted down to reach the final in-well concentration of 2×10^5^ CFU per mL. The suspension was subjected to a 100-fold dilution in Muller–Hinton broth (MHB).

The minimum inhibitory concentration (MIC) and half-maximal inhibitory concentration (IC_50_) values were determined using broth microdilution protocols.^15^ Assays were conducted in 96-well microtiter plate with a total volume of 225 µL per well, testing compounds across a concentration range of 15.6–500 µg mL^−1^. Plates were incubated at 37 °C for 18–24 h prior to analysis. The MIC was defined as the lowest concentration that completely prevented visible growth.^16^ To model the full continuous dose–response relationship across sub-inhibitory concentrations, relative growth inhibition was calculated by normalizing sample optical density against the positive growth control (OD_sample_/OD_positive control_). IC_50_ values were determined by fitting the normalized dose–response data to a 4-Parameter Logistic (4PL) regression model by MyCurveFit. All assays were conducted as three independent biological replicates **(***N* = 3**)**, with each condition tested in technical triplicates.

## 3 Results and discussion

### 3.1 Particle size, morphology, and fractional group of Cu–CuO/Cu_2_O NCs analysis

The Cu–CuO/Cu_2_O NC crystal structure and size were confirmed using XRD investigation. The structure pattern is shown in Fig. 3. The Cu^0^–copper metallic phase is linked with peaks around 2*θ* = 42.5° (111), 52.7° (200), and 73.7° (220), which corresponds directly with the FCC face-centered cubic structure and JCPDS card number (85-1326). Add that the CuO (copper(ii) oxide) phase was linked to peaks round 2*θ* = 35.7° (111), 38.8° (200), and 49.02° (202) corresponding to JCPDS card number (80-1916), and the Cu_2_O (copper(i) oxide) phase was matched with peaks 2*θ* = 29.7° (110), 36.6° (111), 61.5° (220), and 77.6° (222), corresponding directly with the JCPDS card number (75.1531).^5,17^ The (111) planes, which presented with 2*θ* values of 42°, aligning with the mixed phase powders Cu_2_O, CuO, and Cu matched with JCPDS card index 75-1525.^5^

**Fig. 3:**
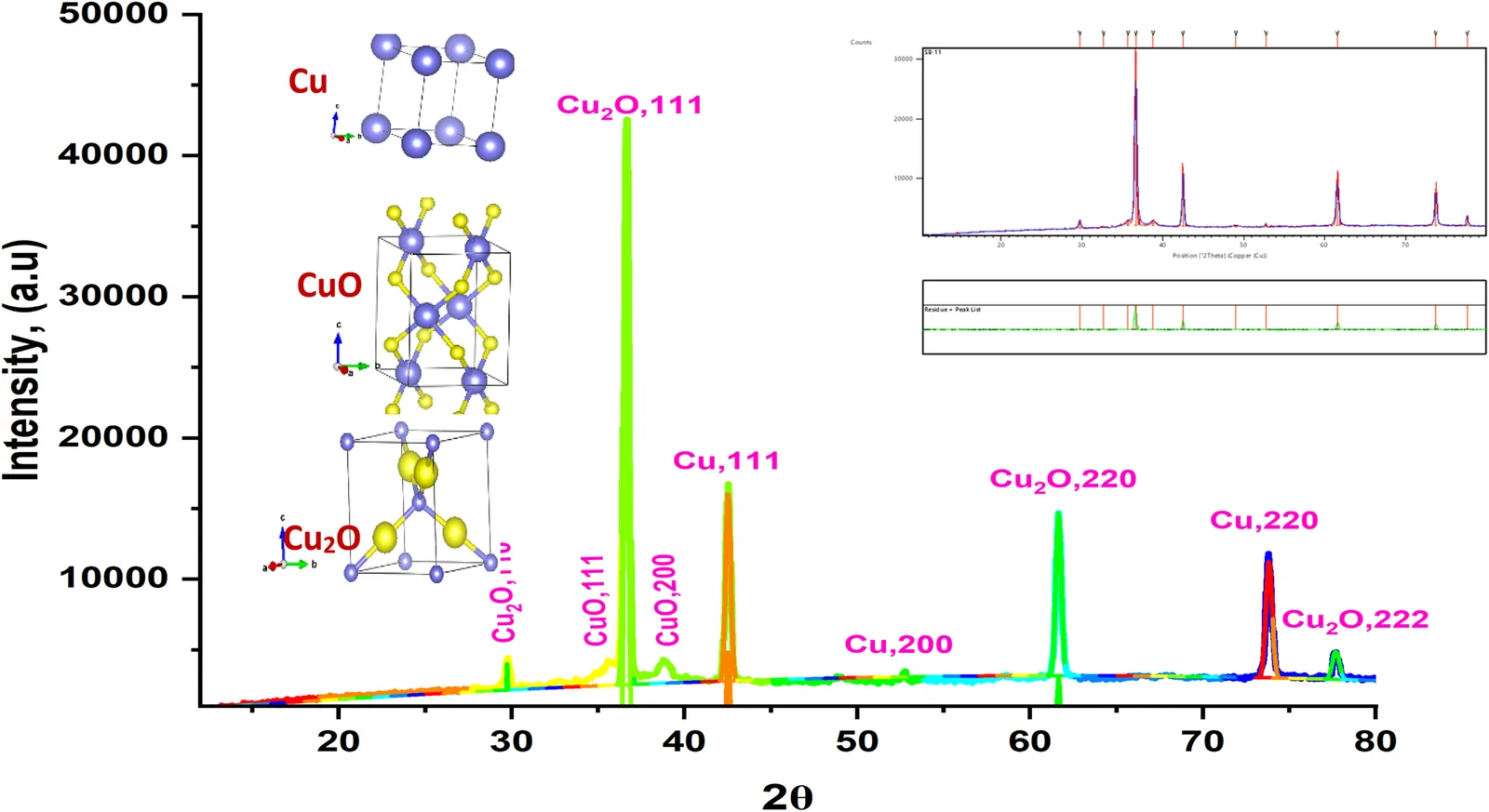
XRD pattern for Cu–CuO/Cu_2_O nanocomposite.

The crystallinity and purity of Cu–CuO/Cu_2_O NCs were demonstrated by the sharp and well-defined peaks in the XRD pattern shown in Fig. 3.

The “Debye–Scherrer formula” in eqn (1) was utilized with results from HighScorePlus software, which automatically identified peaks, determining the average particle size of the Cu–CuO/Cu_2_O NCs to be around 21.3 nm; Table 2 presented the XRD results.

XRD intensity peaks, 2-theta, crystal size, Miller index values, and phase of the Cu–CuO/Cu_2_O NCs

**Table:** 

Pos. [°2Th.]	FWHM	Particle size, (nm)	Miller values	Phase
29.7118	0.3	25.31141	(110)	Cu_2_O
35.6666	0.48	15.75537	(111)	CuO
36.662	0.3	25.1372	(111)	Cu_2_O
38.804	0.72	10.40714	(200)	CuO
42.4751	0.24	30.85319	(111)	Cu^0^, CuO, Cu_2_O
49.0201	0.72	10.03947	(202)	CuO
52.7264	0.18	39.54478	(200)	Cu^0^
61.5453	0.36	18.96046	(220)	Cu_2_O
73.705	0.3	21.18959	(220)	Cu0
77.6058	0.24	25.79598	(222)	CuO

**Table:** 

Descriptive statistics							
SD for mean	*N* total	Mean	SD	Sum	Minimum	Median	Maximum
	10	21.23486	9.33557	233.5835	10.03947	21.18959	39.54478

The morphology of Cu–CuO/Cu_2_O NCs before and after loading OTC on Cu–CuO/Cu_2_O NCs was illustrated in Fig. 4. Fig. 4A and B showed pure Cu–CuO/Cu_2_O NCs in 1 µm and 200 nm zoom before delivery with the drug. Cu–CuO/Cu_2_O NCs morphology was a homogeneous fine granular particle, highly agglomerated due to the incomplete crystallization process, featuring an average grain diameter of 29.52 nm with a range of 5–53, as seen in Fig. 4C. Fig. 4D and E illustrate Cu–CuO/Cu_2_O nanocomposites after loading with the OTC antibiotic at magnifications of 1 µm and 200 nm, respectively. The results presented in Fig. 4D and E of the SEM images confirm the successful delivery of Cu–CuO/Cu_2_O nanocomposites with the OTC drug, indicating that the drug delivery process was successful.

**Fig. 4:**
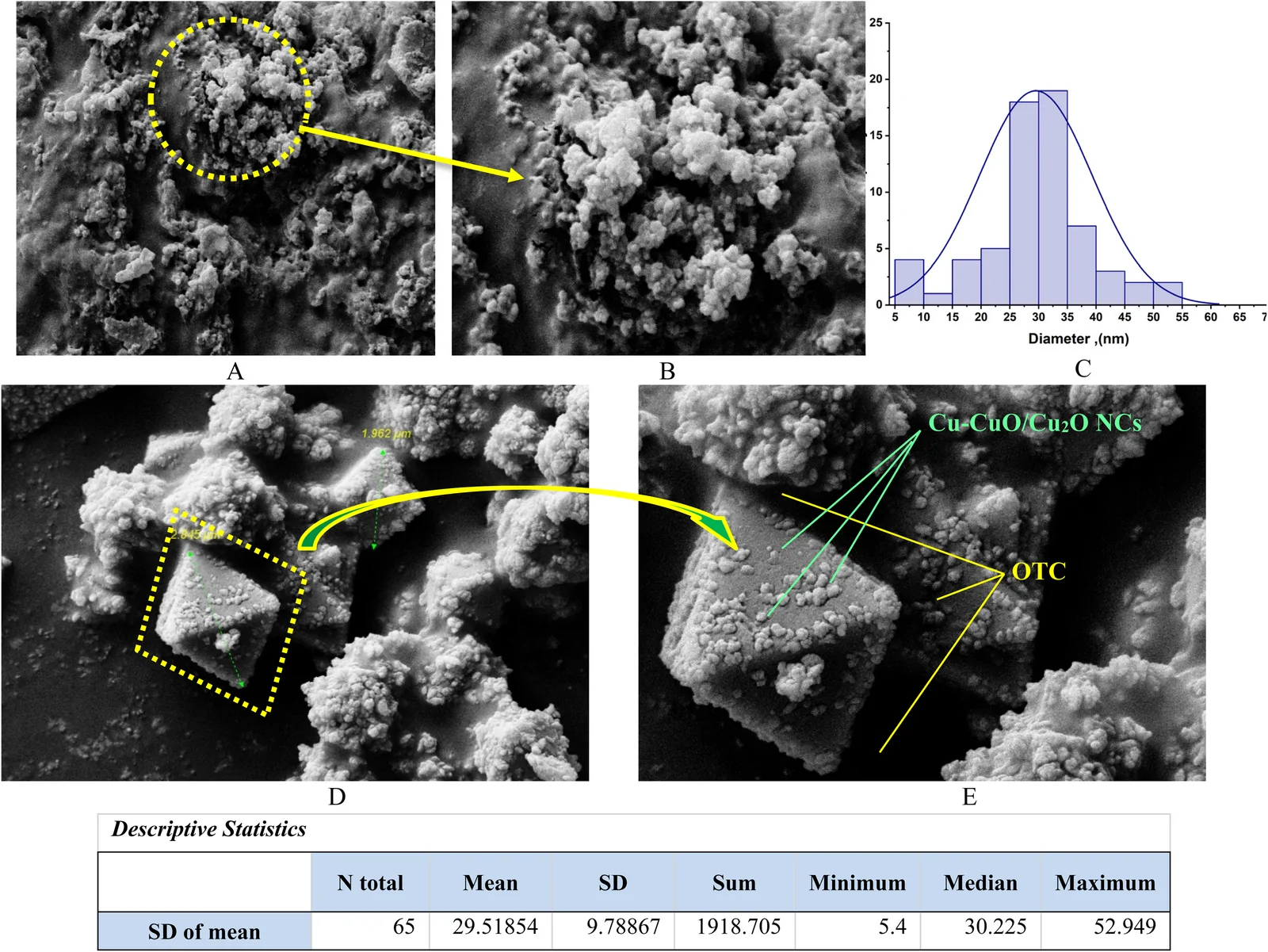
Cu–CuO/Cu_2_O nanocomposite SEM images: 4 (A–C) before OTC delivery, and 4 (D and E) after OTC delivery.

Additionally, the elemental mapping percentage and energy dispersive X-ray (EDX) spectra for the elements Cu and O in Cu–CuO/Cu_2_O NCs were recorded to determine the product's composition, as seen in Fig. 5. The EDX mapping of Cu–CuO/Cu_2_O NCs was illustrated in Fig. 5A–D; they showed homogenous analysis of the distribution of copper and oxygen elements in Cu–CuO/Cu_2_O NCs. EDX spectra verify the existence of elements like Cu and O, and the elements’ percentage in Fig. 5E shows the composite is free of impurities.^18^

**Fig. 5:**
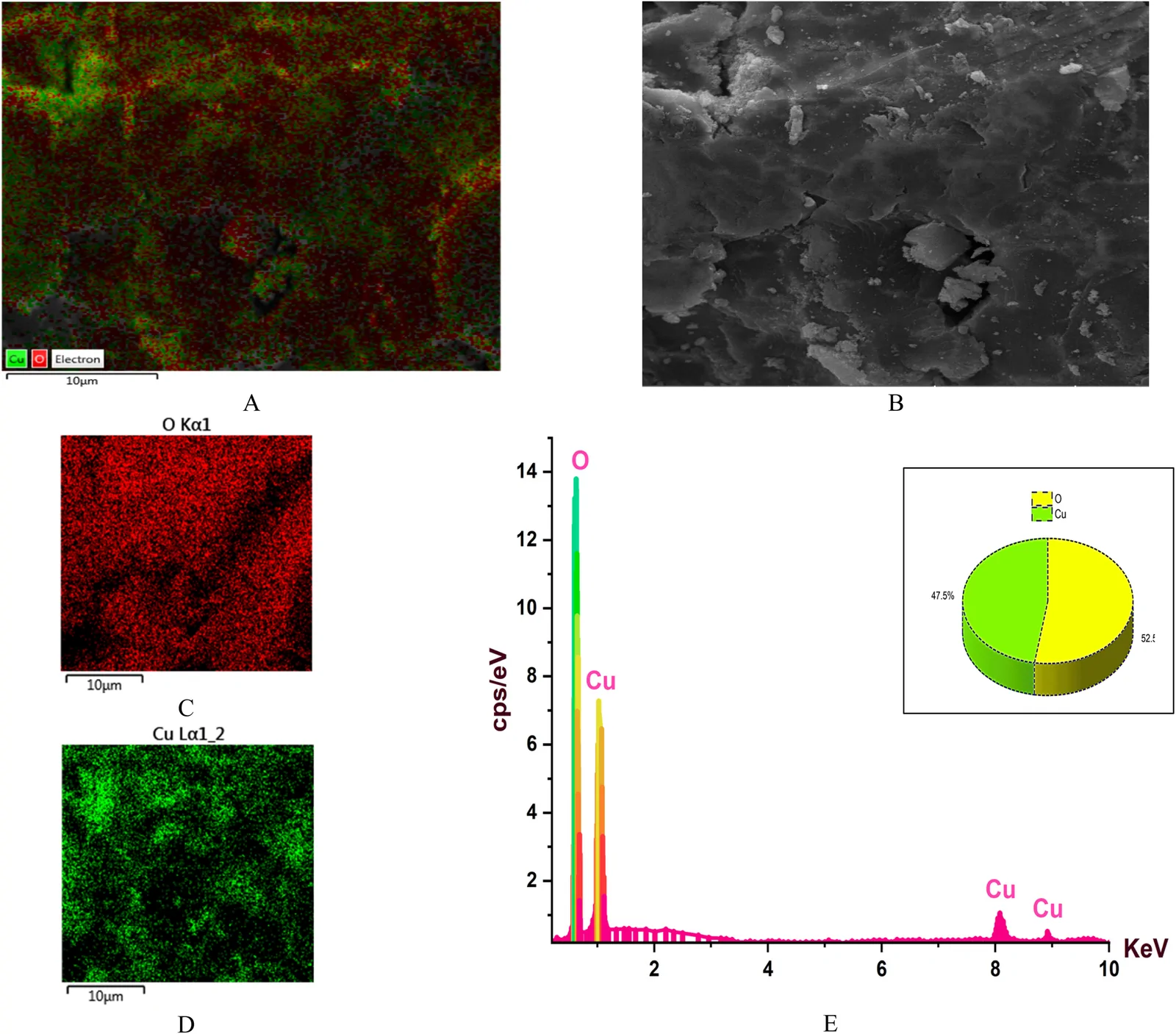
(A–D) EDX mapping spectra of Cu–CuO/Cu_2_O NCs, (E) EDX spectra images of Cu–CuO/Cu_2_O NCs.

Fourier-transform infrared spectroscopy (FT-IR) is used to determine a molecule's functional groups. It also confirms the synthesis of Cu–CuO/Cu_2_O NCs. Fig. 6 depicts how each chemical bond has its own power absorption band.

**Fig. 6:**
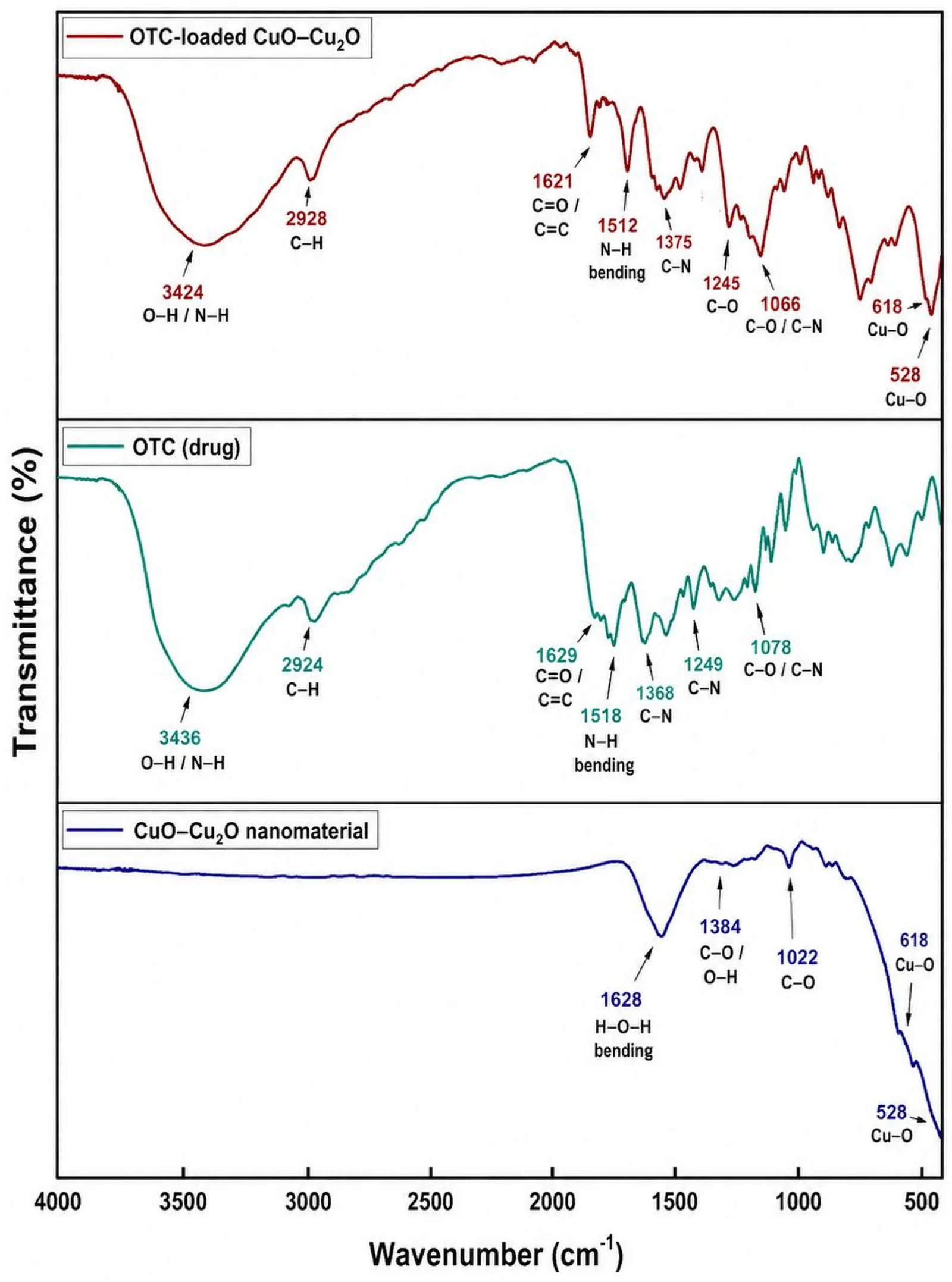
FT-IR analysis of Cu–CuO/Cu_2_O NCs, OTC (drug), and OTC-loaded Cu–CuO/Cu_2_O.

Fig. 6 shows FTIR spectra for pure OTC, Cu–CuO/Cu_2_O NCs, and OTC-loaded Cu–CuO/Cu_2_O NCs. The spectrum of pure OTC contains numerous distinct absorption bands, indicating the presence of oxygen- and nitrogen-containing functional groups. The broad band centered at 3436 cm^−1^ is primarily due to O–H stretching, with a possible contribution from N–H stretching. The band around 2924 cm^−1^ corresponds to C–H stretching vibrations. The absorption at 1629 cm^−1^ is attributed to carbonyl/ring vibrations and may overlap with N–H bending vibrations, corresponding with published FTIR properties of oxytetracycline.^19^

OTC bands at 1518, 1368, 1249, and 1078 cm^−1^ correspond to C=C, C–N, and C–O functional groups. Because many OTC functional groups absorb in the same spectral region, these assignments should be interpreted as overlapping contributions rather than evidence of a single isolated bond.

The spectrum of the Cu–CuO/Cu_2_O nanocomposite is rather simple in the high-wavenumber region, while discrete bands arise in the low-wavenumber region. The bands between 528 and 618 cm^−1^ correspond to metal–oxygen vibrations, which are consistent with Cu–O vibrations from copper oxide phases. FTIR investigations of Cu–CuO/Cu_2_O nanocomposites typically show Cu–O vibrations in the 500–700 cm^−1^ range.^20^

The spectrum of the OTC-loaded Cu–CuO/Cu_2_O nanocomposite reflects characteristics of both components. The bands at 3424, 2928, 1621, 1512, 1375, 1245, and 1066 cm^−1^ correspond to OTC functional groups, while the bands at 618 and 528 cm^−1^ are unique to the copper–oxygen framework. Small shifts in the characteristic bands of pure OTC and loaded material, such as from 3436 to 3424 cm^−1^ and 1629 to 1621 cm^−1^, along with changes in band intensity, indicate a contact between OTC and the nanomaterial surface.

### 3.2 Antimicrobial activity of Cu–CuO/Cu_2_O NCs

One of the 21st century's major issues is the emergence and prevalence of antimicrobial-resistant microbes. In recent decades, the importance of the time it takes to cure illnesses caused by pathogenic microbes has grown. Antimicrobial nanometals and metal oxides as inorganic materials, offer a viable resolution to this matter.^1^ The minimum inhibitory concentration (MIC) method was utilized to evaluate the effectiveness of Cu–CuO/Cu_2_O NCs as antimicrobial agents against a range of bacterial strains, including both Gram-negative and Gram-positive, as well as fungal organisms. Determining the minimum inhibitory concentration (MIC) is crucial for the effective application of antibacterial or antifungal treatments. MIC detection serves as a commonly employed assay that, while fundamentally qualitative, is not suitable for statistical analyses. Therefore, we also utilized half maximal inhibitory concentration (IC_50_) detection, which is a quantitative method that allows us to calculate standard deviation.^15^

This research presents findings from Fig. 7–9, illustrating that the minimum inhibitory concentration (MIC) of the OTC drug against the Gram-negative *E. coli* bacteria is 125 µg mL^−1^, accompanied by an IC_50_ of 64 µg mL^−1^. Moreover, the minimum inhibitory concentration (MIC) for Cu–CuO/Cu_2_O nanocomposites against *E. coli* is documented at 125 µg mL^−1^, alongside an IC_50_ value of 47.1 µg mL^−1^. Furthermore, the minimum inhibitory concentration for OTC@Cu–CuO/Cu_2_O is recorded at 62 µg mL^−1^, accompanied by an IC_50_ value of 29.4 µg mL^−1^.

**Fig. 7:**
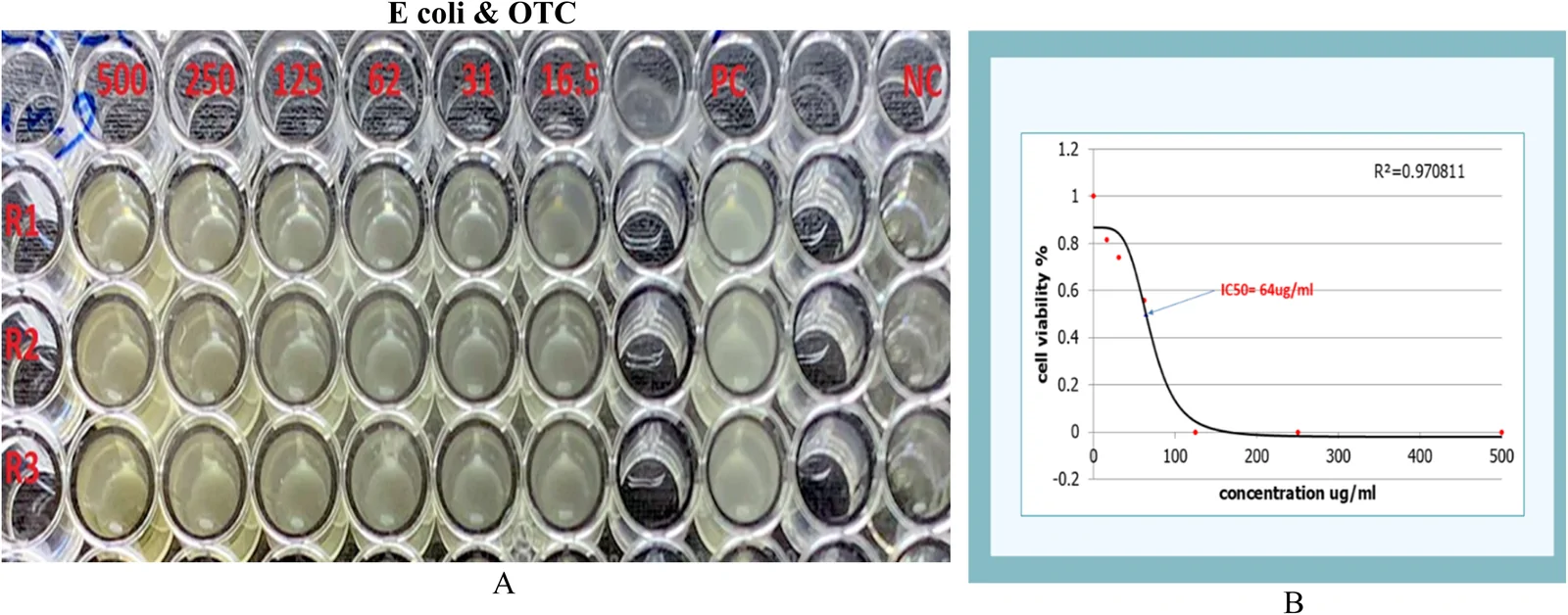
(A) Inhibitory assay for *E. coli*; the microplate illustrates the treatment of *E. coli* with varying concentrations of OTC *R*^1^, *R*^2^, and *R*^3^ replicas. PC serves as the positive control, while *N*_c_ functions as the negative control. Minimum Inhibitory Concentration (MIC) is 125 µg mL^−1^. (B) Optical density of cells *versus* OTC concentration for estimating the IC_50_ value.

**Fig. 8:**
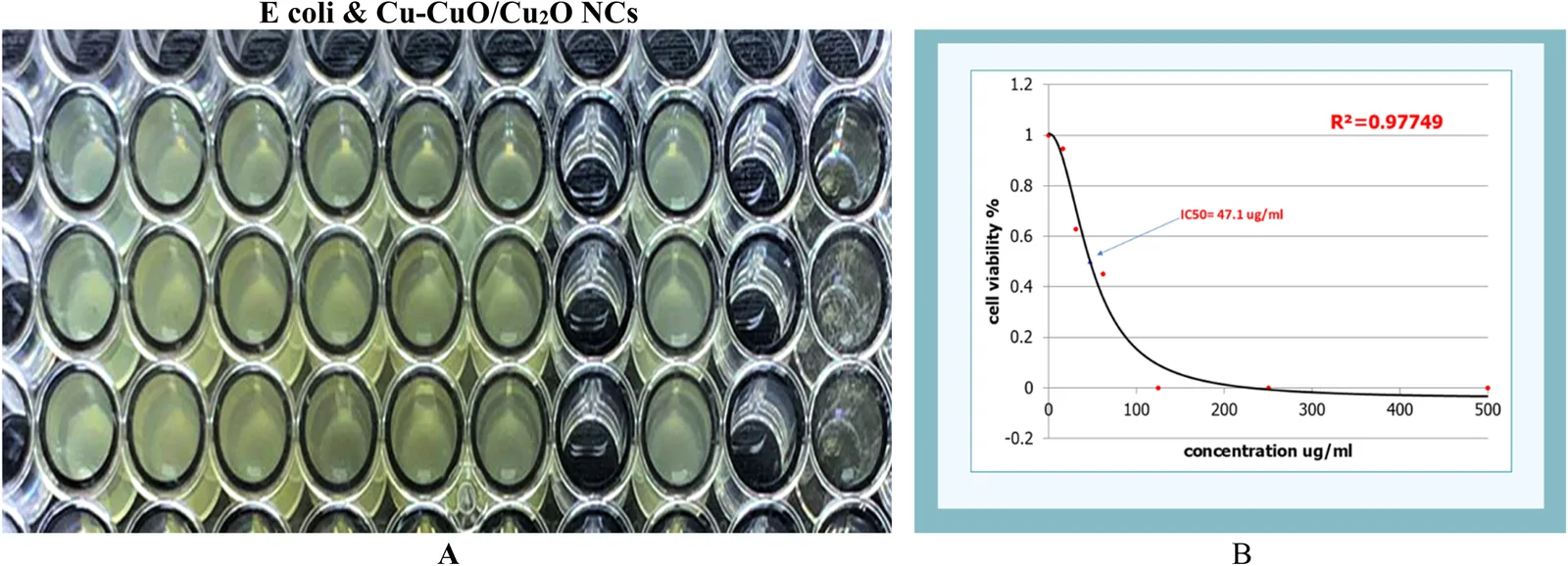
(A) Inhibitory assay for *E. coli*, the microplate illustrates the treatment of *E. coli* with varying concentrations of Cu–CuO/Cu_2_O nanocomposites *R*^1^, *R*^2^, and *R*^3^ replicas. PC serves as the positive control, while *N*_c_ functions as the negative control. Minimum Inhibitory Concentration (MIC) is: 125 µg mL^−1^. (B) Optical density of cells *versus* Cu–CuO/Cu_2_O NCs concentration for estimating the IC_50_ value.

**Fig. 9:**
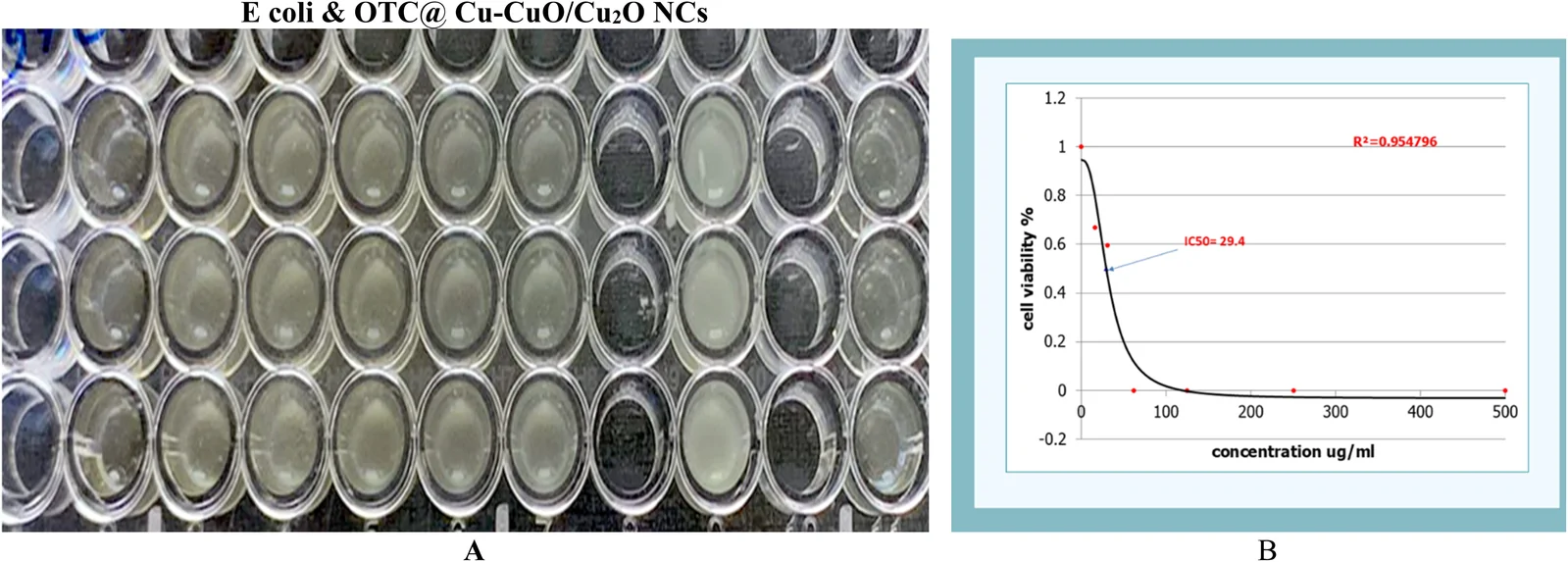
(A) Inhibitory assay for *E. coli*, the microplate illustrates the treatment of *E. coli* with varying concentrations of OTC@Cu–CuO/Cu_2_O nanocomposites *R*^1^, *R*^2^, and *R*^3^ replicas. PC serves as the positive control, while *N*_c_ functions as the negative control. Minimum Inhibitory Concentration (MIC) is: 62 µg mL^−1^. (B) Optical density of cells *versus* OTC@Cu–CuO/Cu_2_O NCs concentration for estimating the IC_50_ value.

Fig. 10–12 demonstrate that the minimum inhibitory concentration (MIC) of the OTC drug against Gram-positive *Staphylococcus aureus* is 250 µg mL^−1^, accompanied by an IC_50_ of 49.2 µg mL^−1^. Conversely, the minimum inhibitory concentration for Cu–CuO/Cu_2_O nanocomposites against *Staphylococcus aureus* was determined to be 500 µg mL^−1^, accompanied by an IC_50_ value of 217 µg mL^−1^. Furthermore, the MIC for OTC@Cu–CuO/Cu_2_O was determined to be 62 µg mL^−1^, demonstrating an IC_50_ of 21.8 µg mL^−1^.

**Fig. 10:**
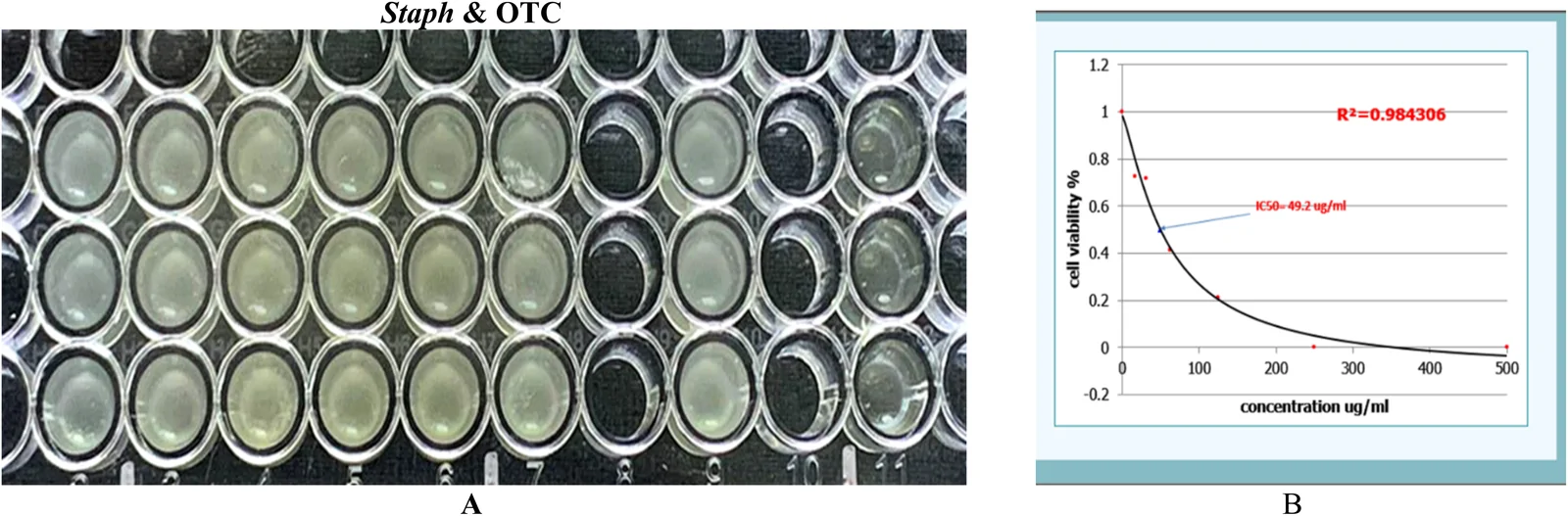
(A) Inhibitory assay for *Staph. aureus*, the microplate illustrates the treatment of *Staph. aureus* with varying concentrations of OTC, *R*^1^, *R*^2^ and *R*^3^ replicas. PC serves as the positive control, while *N*_c_ functions as the negative control. Minimum Inhibitory Concentration (MIC) is: 250 µg mL^−1^. (B) Optical density of cells *versus* OTC concentration for estimating the IC_50_ value.

**Fig. 11:**
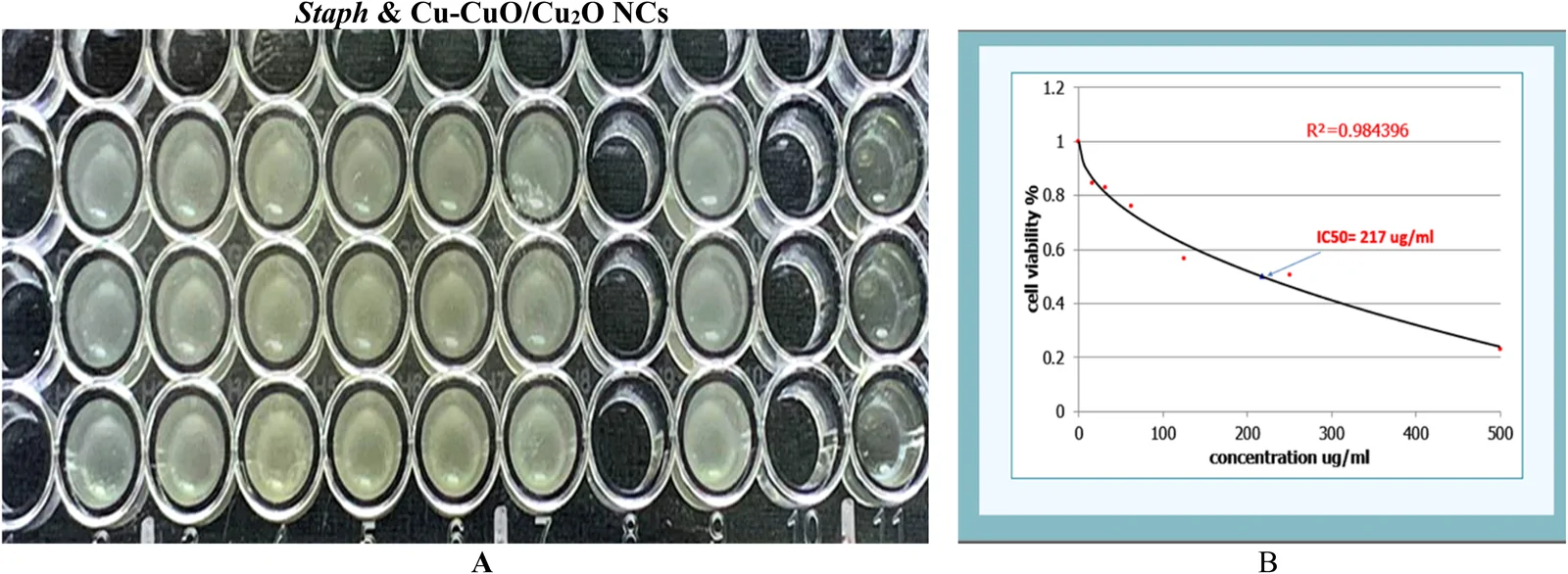
(A) Inhibitory assay for *Staph. aureus*, the microplate illustrates the treatment of *Staph. aureus* with varying concentrations of Cu, *R*^1^, *R*^2^ and *R*^3^ replicas. PC serve as the positive control, while *N*_c_ functions as the negative control. Minimum Inhibitory Concentration (MIC) is >500 µg mL^−1^. (B) Optical density of cells *versus* Cu–CuO/Cu_2_O NCs concentration for estimating the IC_50_ value.

**Fig. 12:**
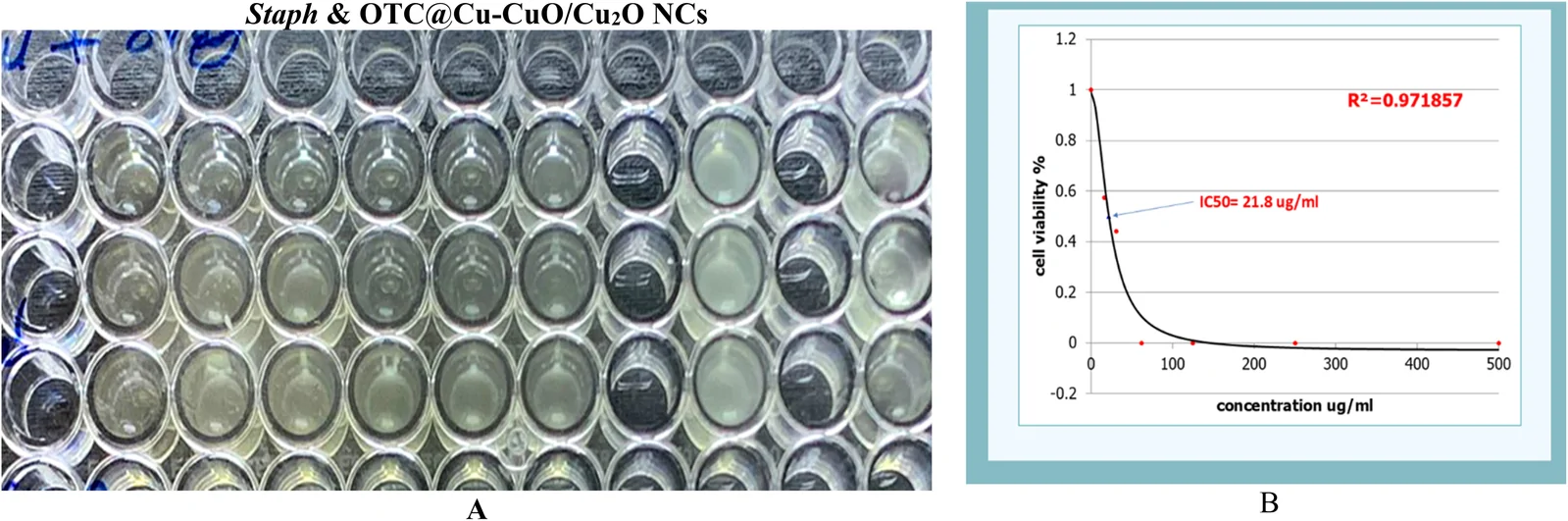
(A) Inhibitory assay for *Staph. aureus*; the microplate illustrates the treatment of *Staph. aureus* with varying concentrations of OTC@Cu–CuO/Cu_2_O, *R*^1^, *R*^2^, and *R*^3^ replicas. PC serves as the positive control, while *N*_c_ functions as the negative control. Minimum Inhibitory Concentration (MIC) is 62 µg mL^−1^. (B) Optical density of cells *versus* of OTC@Cu–CuO/Cu_2_O concentration for estimating the IC_50_ value.

In Fig. 13–15, the minimum inhibitory concentration (MIC) of the over-the-counter medication against *Candida albicans* is recorded at 500 µg mL^−1^, accompanied by an IC_50_ value of 125 µg mL^−1^. Conversely, the minimum inhibitory concentration for Cu–CuO/Cu_2_O nanocomposites against *Candida albicans* exceeds 500 µg mL^−1^, presenting an IC_50_ value of 486 µg mL^−1^. Furthermore, the minimum inhibitory concentration for OTC@Cu–CuO/Cu_2_O is established at 250 µg mL^−1^, resulting in an IC_50_ value of 37.5 µg mL^−1^.

**Fig. 13:**
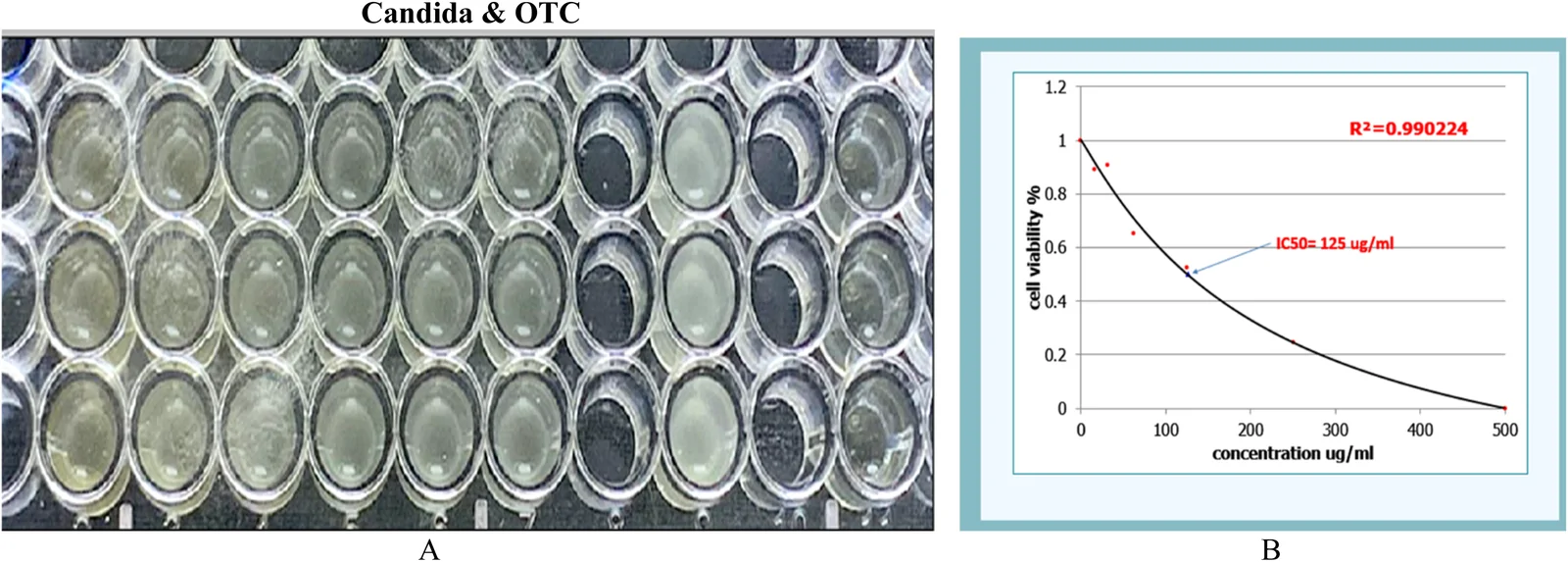
(A) Inhibitory assay for *Candida albicans*, the microplate illustrates the treatment of *Candida albicans* with varying concentrations of OTC, *R*^1^, *R*^2^, and *R*^3^ replicas. PC serves as the positive control, while *N*_c_ functions as the negative control. Minimum Inhibitory Concentration (MIC) is: 500 µg mL^−1^. (B) Optical density of cells *versus* OTC concentration for estimating the IC_50_ value.

**Fig. 14:**
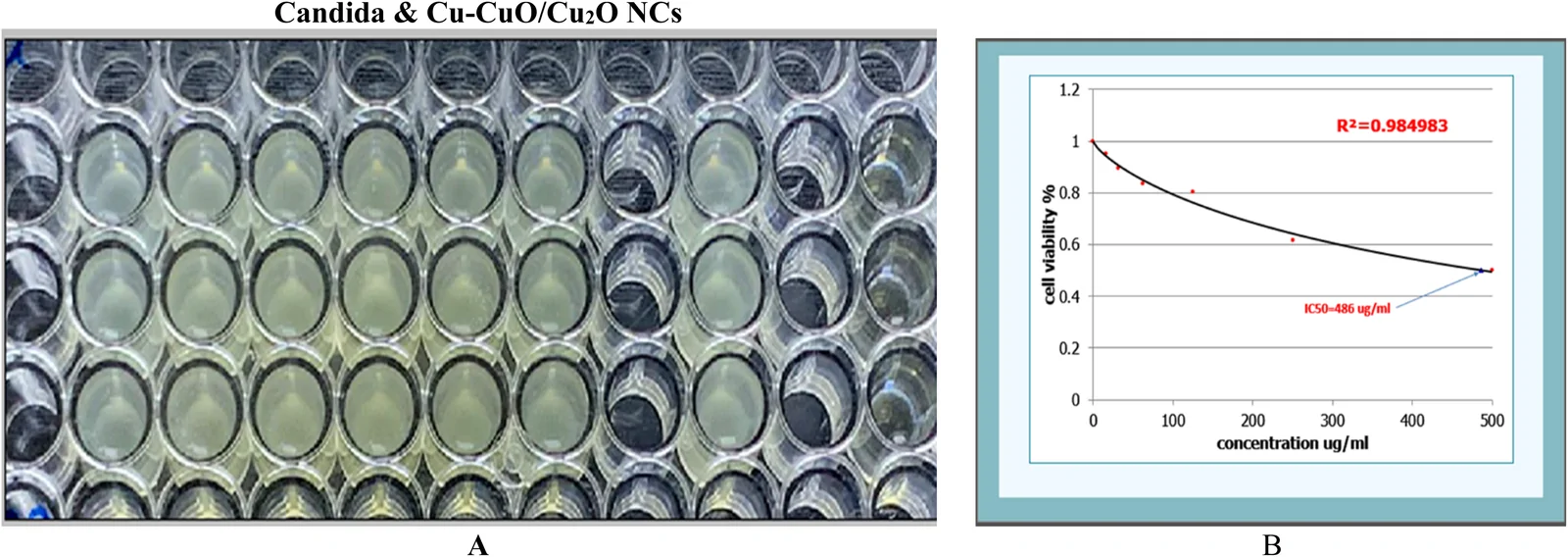
(A) Inhibitory assay for *Candida albicans*, the microplate illustrates the treatment of *Candida albicans* with varying concentrations of Cu–CuO/Cu_2_O NCs, *R*^1^, *R*^2^, and *R*^3^ replicas. PC serve as the positive control, while *N*_c_ functions as the negative control. Minimum Inhibitory Concentration (MIC) is >500 µg mL^−1^. (B) Optical density of cells *versus* Cu–CuO/Cu_2_O NCs concentration for estimating the IC_50_ value.

**Fig. 15:**
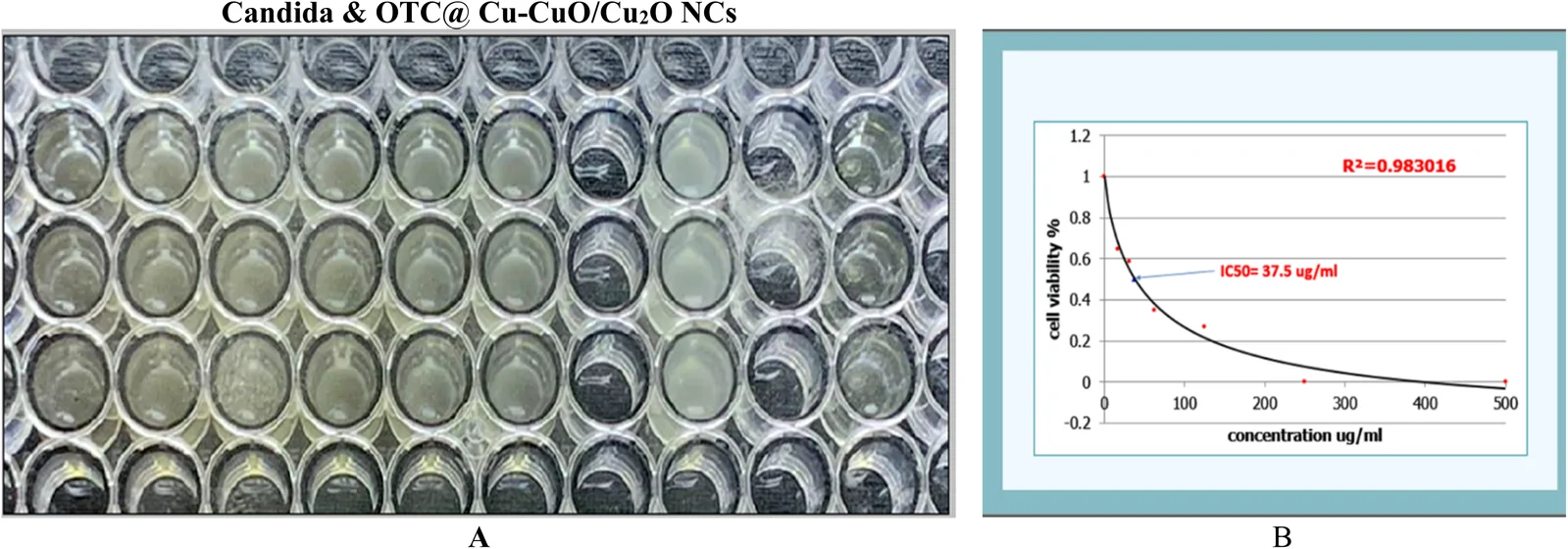
(A) Inhibitory assay for *Candida albicans*, the microplate illustrates the treatment of *Candida albicans* with varying concentrations of OTC@Cu–CuO/Cu_2_O NCs *R*^1^, *R*^2^, and *R*^3^ replicas. PC serve as the positive control, while *N*_c_ functions as the negative control. Minimum Inhibitory Concentration (MIC) is 250 µg mL^−1^. (B) Optical density of cells *versus* OTC@Cu–CuO/Cu_2_O NCs concentration for estimating the IC_50_ value.

The results demonstrated that the OTC@Cu–CuO/Cu_2_O exhibited a significantly greater impact on the Gram-negative bacterium *E. coli* in comparison to the Gram-positive bacteria *Staphylococcus aureus* and *Candida albicans*. The Cu–CuO/Cu_2_O NCs, when combined with the pharmaceutical agent, demonstrated enhanced effectiveness against Gram-positive bacteria in comparison to *Candida albicans*. Oxytetracycline (OTC) when delivered *via* Cu–CuO/Cu_2_O NCs, can be attributed to a multi-targeted multi-targeted mechanism against *Escherichia coli*, *Staphylococcus aureus*, and *Candida albicans*. Structural characterization reveals that the heterogeneous phase composition and high surface area of the Cu–CuO/Cu_2_O matrix provide excellent loading sites, facilitating a sustained and controlled release of OTC directly at the microbial site.^21^ For the bacterial strains, the NCs act as an effective carrier that destabilizes the microbial cell envelopes. In Gram-negative *E. coli*, the nano-carrier helps bypass the complex outer membrane barrier, while in Gram-positive *S. aureus*, the localized release of copper ions induces severe membrane depolarization and lipid peroxidation. Simultaneously, the mixed-valence states of copper trigger the intracellular generation of Reactive Oxygen Species (ROS) *via* Fenton-like reactions, leading to protein denaturation and irreversible DNA damage.^22^ This oxidative stress works in tandem with OTC, which enters the compromised cells to inhibit protein synthesis, effectively overwhelming the bacterial defense mechanisms. Furthermore, this enhanced antimicrobial activity extends to the fungus *C. albicans*, where the copper species mechanically disrupt the chitin-rich fungal cell wall, allowing OTC—which typically lacks potent standalone antifungal properties—to exert structural and cellular stress, ultimately resulting in enhanced microbial growth inhibition.^23^

Ardalani *et al.* and Shabatina *et al.* identified the additive effect of Cu-NPs in combination with erythromycin, azithromycin, and norfloxacin against Gram-positive bacteria (*Staphylococcus* spp.) and Gram-negative bacteria (*Escherichia coli*, *Klebsiella* spp., *Shigella* spp., and *Pseudomonas* spp.) utilizing the disc diffusion assay method. The hybrid Dioxidine@Cu-NPs exhibited a superior antibacterial effect compared to the cumulative impact of the individual components. Cephalexin@CuO exhibited improved antibacterial effectiveness against *E. coli*.

Nanometals can be tailored to align with the specific characteristics of receptors found on the cell surface. The enhancement of these nanocomposites can be effectively accomplished *via* the utilization of polymer coatings. Both functional groups and polymer systems demonstrate considerable efficacy as binding and retention agents for nanoparticles, particularly in antibacterial applications. The utilization of dendrimers within a targeted delivery system signifies a plausible resolution. Pharmaceutical dendrimers demonstrate antibacterial characteristics and function as both a pivotal link and an active element within these systems. Metallic copper (Cu), cupric oxide (CuO), cuprous oxide (Cu_2_O) nanoparticles, and Cu_2_O/CuO composites, commonly referred to as copper-based nanoparticles, have garnered interest due to their antibacterial characteristics and cost-effectiveness.^1^

Metal nanoparticles primarily inflict harm on bacterial cells through the oxidative cellular stress that is instigated by reactive oxygen species (ROS). Reactive oxygen species (ROS) include free radicals such as hydroxyl radicals (˙OH), superoxide radicals (O_2_˙^−^), and hydroperoxyl radicals (HO_2_˙ or HOO˙), along with nonradical forms like singlet oxygen (O_2_) and hydrogen peroxide (H_2_O_2_). Reactive oxygen species (ROS) serve as essential intermediaries in various physiological processes, including photosynthesis, respiration, and cellular signaling, with their intercellular concentrations being precisely regulated by enzymes. Metal ions catalyze an excessive generation of reactive oxygen species, resulting in the degradation of organelle architectures and biomolecular integrity. The overproduction of reactive oxygen species (ROS) contributes to the oxidative carbonylation of proteins, disrupts membrane integrity, induces lipid peroxidation, and compromises the integrity of DNA and RNA, ultimately resulting in heightened cell necrosis, apoptosis, or potential mutations.^1,24^

Apoptosis, predominantly of mitochondrial origin, has been identified as a vital mechanism of cellular demise instigated by oxidative stress resulting from metal nanoparticles. Increased levels of reactive oxygen species within the mitochondria may lead to detrimental effects on membrane phospholipids and cause membrane depolarization. A range of metal oxide nanoparticles, including copper, zinc, titanium oxides, and silicon dioxide, induce ROS-mediated cellular apoptosis through the mechanism of mitochondrial dysfunction.^25^

Comparison of antimicrobial activity across all test groups clearly demonstrated that loading oxytetracycline (OTC) onto the Cu–CuO/Cu_2_O nanocomposites substantially enhanced its efficacy. MIC values were markedly reduced relative to the free drug alone: from 125 µg mL^−1^ to 62 µg mL^−1^ for *E. coli*, from 250 µg mL^−1^ to 62 µg mL^−1^ for *S. aureus*, and from 500 µg mL^−1^ to 250 µg mL^−1^ for *C. albicans*. In all cases, the OTC@Cu–CuO/Cu_2_O hybrid exhibited lower MIC values than either the free antibiotic or the bare nanocomposite, confirming an enhanced antimicrobial activity.

### 3.3 In summary

Against *S. aureus*, the hybrid demonstrated approximately fourfold greater potency (MIC: 62 µg mL^−1^*vs.* 250 µg mL^−1^ for free OTC). Against *E. coli*, activity was approximately twofold higher (62 µg mL^−1^*vs.* 125 µg mL^−1^), and against *C. albicans*, a twofold improvement was also observed (250 µg mL^−1^*vs.* 500 µg mL^−1^).

The combination of OTC with Cu–CuO/Cu_2_O nanocomposites offers a promising strategy to improve treatment outcomes against resistant pathogens by amplifying the antibiotic's activity through multiple complementary mechanisms, thereby enabling the use of reduced therapeutic doses.

## 4 Conclusion

In this study, a mixed-phase Cu–CuO/Cu_2_O nanocomposite was successfully synthesized *via* chemical reduction. The Cu–CuO/Cu_2_O NCs exhibited a mean particle size of approximately 21.3 nm with XRD, and an average grain diameter (*D*) ≈ 30 nm with a distribution range (5–53), confirming the nanoscale nature of the product. Structural characterization by XRD, SEM, and EDX verified the purity and well-defined composition of the nanocomposite.

Loading oxytetracycline (OTC) onto the nanocomposite yielded OTC@Cu–CuO/Cu_2_O hybrid systems with markedly superior antimicrobial activity compared to free OTC, acting against bacteria and fungi through simultaneous, multi-mechanistic pathways. This multi-target mode of action is expected to significantly reduce the probability of resistance development.

The hybrid system demonstrated strong antimicrobial performance, achieving MIC values as low as 62 µg mL^−1^ against both *E. coli* and *S. aureus*. The observed enhanced antimicrobial activity supports the potential use of reduced antibiotic doses, which could minimize adverse effects and toxicity risks. Overall, these copper-based metal oxide nanocomposites represent promising nanocarriers, owing to their high surface area, ease of surface modification, and demonstrated capacity to enhance the activity of conventional antimicrobials against multidrug-resistant pathogens.

## Author contributions

Asmaa H. Hammadi: investigation (equal), methodology development (equal), writing – original draft (equal), data analysis and interpretation (equal). Saba Abd Ulmunem Habeeb: investigation (equal), writing – original draft (equal), data validation and verification (equal). Lena Fadhil Hamza: formal analysis, methodology development (equal) Rafal Al-Assaly: data curation, data validation and verification (equal). Amer Al-Nafiey: project administration, manuscript review, editing, and overall coherence.

## Conflicts of interest

The authors declare that they have no conflicts of interest.

## Data Availability

The data supporting the findings of this study are available from the corresponding author upon reasonable request.
